# METTL3/N6‐methyladenosine/ miR‐21‐5p promotes obstructive renal fibrosis by regulating inflammation through SPRY1/ERK/NF‐κB pathway activation

**DOI:** 10.1111/jcmm.16603

**Published:** 2021-06-24

**Authors:** Erpeng Liu, Lei Lv, Yonghao Zhan, Yuan Ma, Jinjin Feng, Yulin He, Yibo Wen, Yanping Zhang, Qingsong Pu, Fengping Ji, Xinghuan Yang, Jian Guo Wen

**Affiliations:** ^1^ Department of Urology First Affiliated Hospital of Zhengzhou University Zhengzhou China; ^2^ Urodynamics Center First Affiliated Hospital of Zhengzhou University Zhengzhou China; ^3^ Henan Joint International Pediatric Urodynamic Laboratory Zhengzhou University Zhengzhou China

**Keywords:** METTL3, miR‐21‐5p, N6‐methyladenosine (m^6^A), renal fibrosis, Spry1/ERK/NF‐κB, urinary tract obstruction

## Abstract

Renal fibrosis induced by urinary tract obstruction is a common clinical occurrence; however, effective treatment is lacking, and a deeper understanding of the mechanism of renal fibrosis is needed. Previous studies have revealed that miR‐21 impacts liver and lung fibrosis progression by activating the SPRY1/ERK/NF‐kB signalling pathway. However, whether miR‐21 mediates obstructive renal fibrosis through the same signalling pathway has not been determined. Additionally, studies have shown that N6‐methyladenosine (m^6^A) modification‐dependent primary microRNA (pri‐microRNA) processing is essential for maturation of microRNAs, but its role in the maturation of miR‐21 in obstructive renal fibrosis has not yet been investigated in detail. To address these issues, we employed a mouse model of unilateral ureteral obstruction (UUO) in which the left ureters were ligated for 3, 7 and 14 days to simulate the fibrotic process. In vitro, human renal proximal tubular epithelial (HK‐2) cells were transfected with plasmids containing the corresponding sequence of METTL3, miR‐21‐5p mimic or miR‐21‐5p inhibitor. We found that the levels of miR‐21‐5p and m^6^A modification in the UUO model groups increased significantly, and as predicted, the SPRY1/ERK/NF‐kB pathway was activated by miR‐21‐5p, confirming that miR‐21‐5p plays an important role in obstructive renal fibrosis by enhancing inflammation. METTL3 was found to play a major catalytic role in m^6^A modification in UUO mice and drove obstructive renal fibrosis development by promoting miR‐21‐5p maturation. Our research is the first to demonstrate the role of the METTL3‐m^6^A‐miR‐21‐5p‐SPRY1/ERK/NF‐kB axis in obstructive renal fibrosis and provides a deeper understanding of renal fibrosis.

## 1 INTRODUCTION

Renal fibrosis is a common pathological change in hydronephrosis that is caused by urinary tract obstruction and leads to renal parenchyma destruction and renal function damage. To date, there is no effective intervention to completely restore renal function and reverse renal fibrosis, even if the obstruction is relieved by surgical treatment. A better understanding of the mechanism underlying progression of obstructive renal fibrosis is necessary to find an effective medicine or treatment procedure. In recent years, the focus of studies on renal fibrosis occurrence and development has gradually shifted from proteins and mRNAs to non‐coding RNAs, including microRNAs, lncRNAs and circRNAs. microRNAs are well studied and widely found in the genomes of animals and plants. Most microRNAs are composed of 21 to 23 nucleotides and affect the mRNA degradation and protein synthesis of numerous genes by binding to specific sites on target genes, thereby participating in a variety of biological processes. MicroRNAs have been demonstrated to play an important role in renal fibrosis development. And miR‐21 is one of the frequently mentioned microRNAs in the field of renal fibrosis; its dysregulation has been found in many renal fibrosis models and clinical samples.^1^, ^2^, ^3^ Most studies agree that functional miR‐21 promotes renal fibrosis,^4^, ^5^, ^6^ but its maturation process and downstream signalling pathway are still unclear and merit further exploration. SPRY1, a direct target of miR‐21, inhibits the ERK/NF‐κB pathway in angiotensin II–induced liver fibrosis^7^ and bleomycin (BLM)‐induced lung fibrosis in rats.^8^ These findings indicate that miR‐21 is involved in the fibrotic process in the lung and liver via the SPRY1/ERK/NF‐kB signalling pathway. However, whether miR‐21 promotes obstructive renal fibrosis via the ERK/NF‐kB signalling pathway by targeting SPRY1 has not been determined.

Mature microRNAs originate from long primary transcripts called pri‐microRNAs. N6‐methyladenosine (m^6^A) modification labels pri‐microRNAs in the nucleus^9^ and is recognized and bound by nuclear reader protein, which recruits DGCR8 and the nuclear RNase III DROSHA to cleave the stem loop and produce pre‐microRNAs.^10^ Then, pre‐microRNAs are exported to the cytoplasm for further splicing. The m^6^A modification is the most abundant RNA modification in eukaryotes and is highly conserved within mRNAs, microRNAs and lncRNAs among many species. In RNA m^6^A modification, a methyl group is added onto the sixth N atom of the RNA base A. This process is catalysed by a core methyltransferase complex consisting of methyltransferase‐like 3 (METTL3), methyltransferase‐like 14 (METTL14) and Wilms tumour 1‐associated protein (WTAP) and is reversed by demethylases, including fat mass and obesity‐associated protein (FTO) and alkB homologue 5 (ALKBH5).^11^, ^12^ Thus, m^6^A RNA modification is a dynamic and reversible process. The reader proteins include YT521‐B homology (YTH) domain family,^13^, ^14^ heterogeneous nuclear ribonucleoproteins (HNRNPs)^14^ and insulin‐like growth factor 2 mRNA‐binding proteins (IGF2BPs).^14^, ^15^ Among them, YTHDC1,^16^ HNRNPA2B1^17^ and HNRNPC^17^ are predominantly found in the nucleus. HNRNPA2B1 has been found to be reader protein of m^6^A in pri‐microRNAs and to promote maturation of microRNAs.^17^ A previous study reported that m^6^A is involved in the metabolism of miRNA‐126 and drives pulmonary fibrosis development.^18^ However, to date, there have been no reports on the role of m^6^A modification in microRNA maturation during obstructive renal fibrosis development.

In the present study, we generated obstructive renal fibrosis models in mice and enhanced or inhibited the expression of miR‐21‐5p, METTL3, HNRNPA2B1 and ERK in HK‐2 cells through transfection or chemical inhibition. We demonstrated that increased miR‐21‐5p levels induced by ureteral obstruction enhanced inflammation of the renal parenchyma by targeting SPRY1 and activating the ERK/NF‐kB pathway, which resulted in extracellular matrix (ECM) deposition and progression of obstructive renal fibrosis. METTL3‐mediated m^6^A modification promoted miR‐21‐5p maturation by promoting recognition and processing of pri‐miR‐21. These findings might provide novel information to further understanding of the mechanism underlying obstructive renal fibrosis.

## 2 MATERIALS AND METHODS

### 2.1 Reagents and antibodies

U0126 (a specific ERK1/2 inhibitor) was purchased from Sigma‐Aldrich (St. Louis, Missouri, USA). Primary antibodies against NF‐κB, p‐NF‐κB, SPRY1, ERK1/2 and p‐ERK1/2 were purchased from Cell Signaling Technology (Massachusetts, USA). Primary antibodies against collagen I, α‐SMA, METTL3, HNRNPA2B1, fibronectin (FN) and β‐actin were purchased from Abcam (Cambridge, USA). Primary antibodies against IL‐6 and TNF‐α were purchased from Proteintech (Wuhan, China). Horseradish peroxidase (HRP)–labelled goat anti‐rabbit secondary antibody was purchased from Sangon Biotech (Shanghai, China). Other reagents are described below.

### 2.2 Animals

Sixty 8‐week‐old female C57BL/6 mice were provided by the experimental animal centre of the Medical College of Zhengzhou University and housed at a 22°C constant room temperature and 47% humidity with a 12‐hours light‐dark cycle and free access to standard laboratory chow and tap water. All experimental procedures on mice were performed in accordance with the National Institutes of Health guidelines and were approved by the Ethical Committee, Animal Care and Use Committee of the First Affiliated Hospital of Zhengzhou University (2020‐KY‐273).

### 2.3 Mouse model of unilateral urethral obstruction (UUO) and experimental groups

A total of 60 mice were used in the current study and were randomly divided into two groups: 30 in the sham operation group and 30 in the UUO group. UUO was performed according to established procedures described in previous studies.^19^ Briefly, each mouse was anaesthetized with inhaled isoflurane, and the left proximal ureter was exposed. Then, the ureter was ligated with 6‐0 silk thread and severed. In the sham operation group, the left ureters of mice were exposed, but not ligated or severed. The 3rd, 7th and 14th days after surgery were the time points for killing. At each time point, a total of 10 mice in the UUO group were executed, and a total of 10 mice in the sham group were also executed to serve as controls. The left kidney specimens were collected, and inferior vena cava blood samples were collected for evaluation of renal function, including blood urea nitrogen (BUN) and serum creatinine (SCr). Part of the kidney tissue was frozen in liquid nitrogen for total RNA and protein extraction, and the remainder was fixed with 4% paraformaldehyde.

### 2.4 Histopathological evaluation

Four per cent paraformaldehyde‐fixed kidney specimens were embedded in paraffin, cut into 4‐μm sections on a rotary microtome (Leica, Heidelberg, Germany) and subjected to Masson's trichrome and haematoxylin and eosin (HE) staining. A Leica DM4B microscope equipped with Leica X software was used to examine the slides and take images. Tubulointerstitial impairment was evaluated according to the scoring criteria reported in a previous study^20^ and included assessment of tubular atrophy, tubular necrosis, lymphocyte infiltration and interstitial fibrosis. The scores for each criterion were as follows: 0 = none; 1 = mild or <25%, 2 = moderate or 25% to 50% and 3 = severe or >50%. The blue Masson staining area was considered to indicate collagen deposition and was analysed using Image‐Pro Plus 6.0 software. Six non‐overlapping fields in each section of the kidney cortex and medulla were selected for scoring or image analysis, and the results are expressed as the means ± SD.

### 2.5 Immunohistochemistry (IHC)

A rotary microtome was used to cut the paraffin‐embedded kidney specimens into 4‐μm sections, which were deparaffinized with xylene and rehydrated using graded ethanol (100%, 95%, 85% and 75%) and distilled water. The sections were incubated with 3% hydrogen peroxide for 10 minutes to block the activity of endogenous peroxidase and then heated with a microwave in 0.01 mol/L citrate buffer (pH 6.0) for 25 minutes for antigen retrieval. The specimens were washed three times with phosphate‐buffered saline (PBS) for 5 minutes each time and then incubated with primary antibodies, including anti‐METTL3 (1:1000), anti‐α‐SMA (1:500), anti‐collagen I (1:500), anti‐TNF‐α (1:300), anti‐IL‐6 (1:200) and anti‐FN (1:1000) antibodies, overnight at 4°C. The next day, after being washed with PBS, the specimens were incubated with HRP‐labelled goat anti‐rabbit secondary antibody for 1 hour at room temperature. Finally, dehydration, clearing, 3,3′‐diaminobenzidine (DAB) staining and neutral resin sealing were performed in sequence. In each section, six non‐overlapping fields of the renal cortex were imaged at high magnification (100×). Image‐Pro Plus 6.0 software was applied to assess the integral optical density (IOD) of the positive area, and the results are expressed as the means ± SD.

### 2.6 Cell culture

The human renal proximal tubular epithelial cell line HK‐2 was purchased from Beina Chuanglian Biotechnology Institute (Beijing, China). HK‐2 cells were cultured in Dulbecco's modified Eagle's medium/nutrient mixture F‐12 (DMEM/F12) supplemented with 10% (v/v) foetal bovine serum (FBS), 100 IU/mL penicillin and 10 mg/mL streptomycin in a humidified atmosphere of 5% CO_2_ at 37°C. The medium was changed every 2 days, and the cells were subcultured before a confluent monolayer could be formed.

### 2.7 Quantitative real‐time polymerase chain reaction (qRT‐PCR)

Total RNA was extracted using TRIzol (Sangon Biotech, B511311, Shanghai, China) and stored at −80°C. A NanoDrop 2000 UV‐Vis spectrophotometer (Thermo Scientific, USA) was used to determine the concentration and quality of total RNA. Then, an M‐MuLV First‐Strand cDNA Synthesis kit (Sangon Biotech, B532435, Shanghai, China) was used for reverse transcription of total RNA. An Applied Biosystems 7500 Sequence Detection System was used to determine the mRNA levels with 2×SG Fast qPCR Master Mix (Low Rox) kit (Sangon Biotech, B639272, Shanghai, China). Each sample was analysed in triplicate. The β‐actin gene served as a control, and the data were analysed using the 2^−(ΔΔCt)^ method. To detect mature miR‐21‐5p or pri‐miR‐21, a microRNA First‐Strand cDNA Synthesis kit (Sangon Biotech, B532453, Shanghai, China) was used to synthesize cDNA following the manufacturer's protocol. A MicroRNAs Quantitation PCR Kit (Sangon Biotech, B532461, Shanghai, China) was used to conduct qRT‐PCR, and an Applied Biosystems 7500 Sequence Detection System was used to detect the levels of miR‐21‐5p and pri‐miR‐21. Each sample was analysed in triplicate. U6 small nuclear RNA served as a control, and the data were analysed using the 2^−(ΔΔCt)^ method. The sequences of all primers are listed in Table 1.

**TABLE 1:** Primers used for reverse transcription and real‐time PCR

Primer name		Sequence
mmu‐α‐SMA	Sense Anti‐sense	CTGTTATAGGTGGTTTCGTGGA GAGCTACGAACTGCCTGAC
mmu‐Collagen I	Sense Anti‐sense	CTTCACCTACAGCACCCTTGTG GATGACTGTCTTGCCCCAAGTT
mmu‐FN	Sense Anti‐sense	TTGTTCGTAGACACTGGAGAC GAGCTATCCAATTTCACCTTCAG
mmu‐U6	Stem loop Sense Anti‐sense	GTCGTATCCAGTGCAGGGTCCGAGGTATTCGCACTGGATACGACAAAATATG CTCGCTTCGGCAGCACA AACGCTTCACGAATTTGCGT
mmu‐miR‐21‐5p	Stem loop Sense Anti‐sense	GTCGTATCCAGTGCAGGGTCCGAGGTATTCGCACTGGATACGACTCAACA ACACTCCAGCTGGGTAGCTTATCAGACTGA TGGTGTCGTGGAGTCG
hsa‐U6	Stem loop Sense Anti‐sense	CGAGCACAGAATCGCTTCACGAATTTGCGTGTCAT CGAGCACAGAATCGCTTCA CTCGCTTCGGCAGCACATAT
hsa‐miR‐21‐5p	Stem loop Sense Anti‐sense	GTCGTATCCAGTGCAGGGTCCGAGGTATTCGCACTGGATACGACACAGCCG CGGCGCAACACCAGTCGATG AGTGCAGGGTCCGAGGTATT
hsa‐pri‐miR‐21	Sense Anti‐sense	CTAACCTCACACTCATCCCATTCT TCTCCTAAACTCTCCTTTTTACACC
mmu‐METTL3	Sense Anti‐sense	ATCCAGGCCCATAAGAAACAAC GATACAGCATCAGTGGGCAAGG
mmu‐METTL14	Sense Anti‐sense	GGAACCTGAGATTGGCAACATAG GTCAGACTTGGATTTGGGAGGAG
mmu‐WTAP	Sense Anti‐sense	AGTTATGGCACGGGATGAGTTA TCCTGCTGTTGCTGCTTTAGTT
mmu‐METTL4	Sense Anti‐sense	GAATGACATGGAGCTTCAAACG CCAGCTTAGGGACAGGCATTCT
mmu‐KIAA1429	Sense Anti‐sense	CAACTGTCTGACCCTGGCAATA TTTTCAGTGTCGGCTGTGGC
mmu‐FTO	Sense Anti‐sense	GACACTTGGCTTCCTTACCTGAC CACCAGGTCCCGAAACAAGC
mmu‐ALKBH5	Sense Anti‐sense	GTGGGACCTTTTGGGTTTCAG GCATACGGCCTCAGGACATTA
mmu‐HNRNPC	Sense Anti‐sense	TTAATGAAAGAAATGCCCGAGC CTCTGCAGCCAGGTTAATATCT
mmu‐ HNRNPA2B1	Sense Anti‐sense	ATCCTGCAAGCAAAAGATCAAG AACAAACAGCTTCTTCACAGTC
mmu‐ YTHDC1	Sense Anti‐sense	GCAAGCAGATCCAGCCAGTCTTC CCCCTCCTTCCTCCTCATTCTCAG
mmu‐β‐actin	Sense Anti‐sense	AGAGGGAAATCGTGCGTGAC CAATAGTGATGACCTGGCCGT

### 2.8 Western blotting (WB) assay

Efficient radioimmunoprecipitation assay (RIPA) tissue/cell lysis buffer (Solarbio, R0010, Beijing, China) was used to isolate total protein from kidney tissues and HK‐2 cells according to the manufacturer's instructions. The total protein concentration was detected with an Enhanced BCA Protein Assay Kit (Beyotime, P0009, Shanghai, China), and 30 µg of total protein was loaded and separated via sodium dodecyl sulphate‐polyacrylamide gel electrophoresis. Then, the proteins in the resolving gel were transferred to a PVDF membrane via electrophoresis in a prepared transfer solution. After blocking with 5% non‐fat milk on a shaker for 2 hours, the membranes were incubated overnight at 4°C with primary antibody against collagen I (1:1000), α‐SMA (1:1000), FN (1:1000), METTL3 (1:1000), HNRNPA2B1(1:1000), Spry1 (1:500), ERK1/2 (1:1000), p‐ERK1/2 (1:1000), NF‐κB (1:500), p‐NF‐κB (1:500), IL‐6 (1:500), TNF‐α (1:500) and β‐actin (1:2000). The membranes were washed in TBST and then incubated with HRP‐labelled goat anti‐rabbit secondary antibody on a shaker for 1 hour at room temperature. After washing with TBST, a Bio‐Rad ChemiDoc™ MP Imaging System and an Omni‐ECL™ Femto Light Chemiluminescence kit (EpiZyme, SQ201, Shanghai, China) were used to visualize the bands. Band densities were quantified with ImageJ software. The β‐actin expression level served as a control.

### 2.9 Cell transfection

The PGV657‐METTL3 plasmid for METTL3 (Gene ID: 56339) overexpression; the PGV102‐shHNRNPA2B1#1 and shHNRNPA2B1#2 plasmid for HNRNPA2B1(Gene ID: 3181) knockdown; the pGV249‐miR‐21‐5p‐inhibitor plasmid and pGV514‐miR‐21‐5p‐mimic plasmid for miR‐21‐5p knockdown and overexpression, respectively; and the corresponding control plasmids were constructed and synthesized by GeneChem (Shanghai, China). HK‐2 cells were transfected with the plasmids using Lipofectamine 3000 reagent (Invitrogen) according to the manufacturer's instructions. The HK‐2 cells were seeded on 6‐well plates at 1 × 10^5^ cells and transfected when the cells reached 60%‐70% confluence. After transfection for 48 hours, the cells were harvested and used in subsequent experiments.

### 2.10 Immunofluorescence

HK‐2 cells (1 × 10^5^) were seeded, allowed to adhere onto glass coverslips in 6‐well plates, washed with PBS, fixed in 4% formaldehyde solution for 30 minutes and then permeabilized with 0.2% Triton X‐100/PBS for 15 minutes. The cells were then blocked with 2% bovine serum albumin in PBS for 30 minutes, incubated with primary antibodies overnight at 4°C, incubated with FITC‐/TRITC‐conjugated secondary antibodies for 1 hour at room temperature and then stained with DAPI. Finally, the cells were observed under a fluorescence microscope.

### 2.11 RNA m^6^A dot blot assays

The poly(A) + RNAs (400 ng) were heated at 55°C for 15 minutes for denaturation and then cooled on ice. Next, the samples were added to a BioDot apparatus (Bio‐Rad, USA) to transfer the RNAs onto a GE Amersham Hybond‐N^+^ membrane (RPN303B, USA). After UV cross‐linking and blocking with 5% non‐fat milk, the membranes were incubated with m^6^A antibody (1:2000, Synaptic Systems, Germany) overnight at 4°C and then incubated with HRP‐conjugated anti‐rabbit IgG (1:3000, Proteintech, Wuhan, China). The Bio‐Rad ChemiDoc™ MP Imaging System and an Omni‐ECL™ Femto Light Chemiluminescence kit (EpiZyme, SQ201, and Shanghai, China) were used to visualize the membranes. To ensure consistency among different groups, the same 400 ng poly(A) + RNAs were transferred onto the membranes and stained with 0.02% methylene blue (Sigma‐Aldrich, M9140) in 0.3 mol/L sodium acetate (pH 5.2).

### 2.12 RNA immunoprecipitation (RIP)

A Magna RIP Kit (Millipore, MA, USA) was used to perform RIP as described in previous studies. Briefly, HK‐2 cells were washed with pre‐cooled PBS, gently scraped off the culture plate and then collected by centrifugation at 1500 rpm for 5 minutes at 4°C. The RIPA lysis buffer provided by the kit was added to lyse the cells. Magnetic beads were incubated with anti‐DGCR8 antibody (Abcam, CA, USA) for 30 minutes at room temperature, thoroughly mixed with lysis buffer and incubated overnight at 4°C. The protein‐RNA complex was digested with proteinase K to purify the RNA. Then, RNA was extracted with phenol:chloroform:isoamyl alcohol (125:24:1) (Solarbio, Beijing, China) and subjected to reverse transcription. Finally, pri‐miR‐21 was analysed via qRT‐PCR. The method used for RNA m^6^A immunoprecipitation was similar to that of used for DGCR8. RNA was extracted from HK‐2 cells to perform RNA m^6^A immunoprecipitation. After digestion with DNase I, the RNA was sonicated for 10 seconds to induce fragmentation. The magnetic beads were incubated with rabbit m^6^A antibody (Abcam, CA, USA) for 1 hour at room temperature, and then, the fragmented RNA, antibody‐magnetic bead complex and RIP buffer were mixed well and incubated overnight at 4°C. After the complex was digested with proteinase K buffer, RNA was purified with phenol:chloroform:isoamyl alcohol (125:24:1), followed by reverse transcription and qRT‐PCR to detect the amount of pri‐miR‐21. IgG antibody served as a negative control.

### 2.13 Statistical analysis

GraphPad Prism 7 was used to determine statistically significant differences. All data are presented as the means ± SD and were analysed using unpaired t tests between two groups and one‐way analysis of variance with Tukey's test for more than two groups. *P* < .05 was considered statistically significant.

## 3 RESULTS

According to the results of ultrasonography and pathological examination (Figures S1 and S2), there was no manifestation of hydronephrosis, kidney damage and collagen deposition in the left kidneys of all the sham‐operated mice. Thus, there is no difference on pathological changes in sham group at three time points for killing. To simplify results, we only listed the data and pictures of sham group at one time point for representing the sham group.

### 3.1 Changes in kidney morphology and impairment of renal function induced by UUO in mice

**FIGURE 1:**
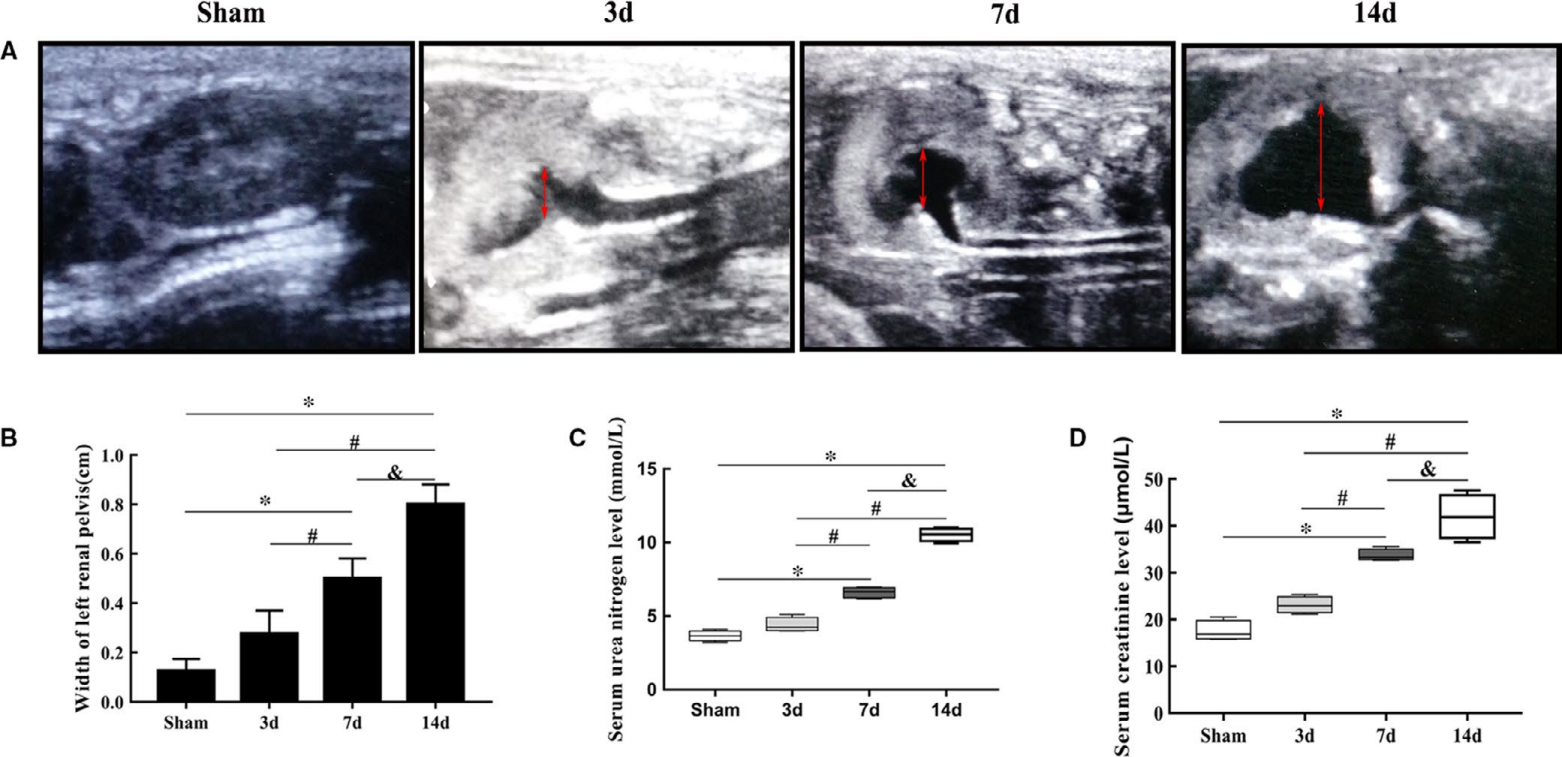
The dramatic change in left kidney morphology under ultrasonography and impairment of renal function. (A and B) The widths of the left renal pelvises in the sham and UUO mouse groups based on ultrasound images (mean ± SD, n = 6). C, BUN levels in the sham and UUO mouse groups. D, SCr levels in the sham and UUO mouse groups. *P < .05, compared to the sham group. ^#^ *P* < .05, compared to the 3‐day UUO group. ^&^ *P* < .05, compared to the 7‐day UUO group. To simplify the figure, we only listed the data and a representative picture of the sham‐operated mice killed on the 7th day after surgery to represent the sham group

After ligation of the left ureters in mice, ultrasonography revealed that the width of the renal pelvis gradually increased, while the renal cortex gradually decreased in thickness (Figure 1A,B). Due to compensation by the right kidney, the BUN and SCr levels were roughly within the normal range (Table 2); however, with the accumulation of urine in the obstructed area, the upward trend within the normal range of BUN and SCr was still obvious (Figure 1C,D).

**TABLE 2:** BUN and SCr levels in the sham and UUO mouse groups (mean ± SD, n = 6)

	Sham 3 days	Sham 7 days	Sham 14 days	UUO 3 days	UUO 7 days	UUO 14 days
BUN (mmol/L)	3.433 ± 0.3311	3.663 ± 0.3572	4.088 ± 0.2333	4.39 ± 0.4956	6.608 ± 0.3517* ^,^ ^#^	10.53 ± 0.4446* ^,^ ^#^ ^,^ ^&^
SCr (μmol/L)	16.899 ± 1.883	17.5 ± 2.153	18.011 ± 1.333	23.06 ± 1.756	33.66 ± 1.278* ^,^ ^#^	41.92 ± 4.707* ^,^ ^#^ ^,^ ^&^

Note

The normal BUN level in mice is 3.86‐12.41 mmol/L. The normal SCr level in mice is 10.91‐85.09 μmol/L.

* *P* < .05, compared to the corresponding sham group.

^#^ *P* < .05, compared to the 3‐day UUO group.

^&^ *P* < .05, compared to the 7‐day UUO group.

### 3.2 Inflammation, collapse of tubular structures and collagen deposition in the renal cortex in UUO mouse models

**FIGURE 2:**
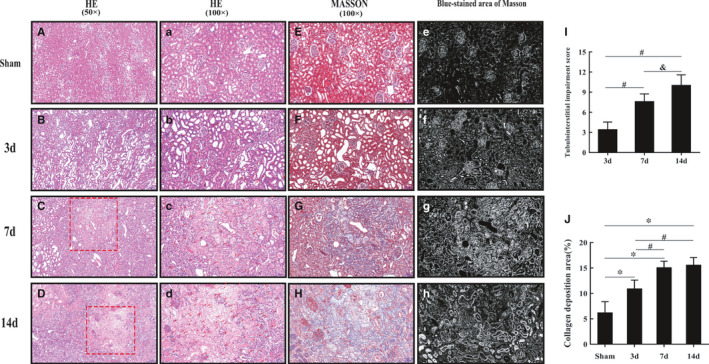
With increased obstruction time, inflammation, parenchymal damage and collagen deposition become increasingly serious in the left renal cortex in the UUO mouse groups. (A‐D and a‐d) Representative histopathology images of obstructed kidneys in mice after HE staining (50× and 100×). Scale bars represent 200 μm and 100 μm. The two red rectangular areas refer to representative inflammatory foci in the 7‐day and 14‐day groups and are amplified in images c and d. The red arrows refer to inflammatory cells in the UUO groups. (E‐H) Representative histopathology images of obstructed kidneys in mice after Masson's trichrome staining (100×). Scale bar represents 100 μm. Images G and H show the same area as images c and d, respectively. (e‐h) Blue Masson's trichrome‐stained areas, which were transformed from image E, F, G and H using Image‐Pro Plus 6.0 software. The white area refers to the collagen deposition area. (I and J). Statistical analyses of tubulointerstitial damage scores and the degree of collagen deposition in different groups (mean ± SD, n = 6). *P < .05, compared to the sham group. ^#^ *P* < .05, compared to the 3‐day UUO group. ^&^ *P* < .05, compared to the 7‐day UUO group. To simplify the figure, we only listed the data and representative pictures of sham‐operated mice killed on the 7th day after surgery to represent the sham group

HE staining showed that the proximal renal tubules were significantly dilated, which worsened over time. Inflammatory cells accumulated, especially in the 7‐day and 14‐day groups, and inflammatory foci (Figure 2C,D,c,d) were formed with massive inflammatory cell infiltration. The structure of renal tubules was damaged, and many glomeruli collapsed (Figure 2A‐D,a‐d,I). The blue Masson's trichrome‐stained area, which reflected collagen deposition in renal tissue, increased in a time‐dependent manner compared with that in the sham group (Figure 2E‐H,e‐h,J). We also found more obvious blue Masson's trichrome staining in the area of inflammatory foci (Figure 2G,H,g,h), and the expression of IL‐6 and TNF‐α increased significantly in the 7‐day and 14‐day groups (Figure 4B‐M).

### 3.3 The expression of fibrosis indicators increased in a time‐dependent manner in UUO mouse models

**FIGURE 3:**
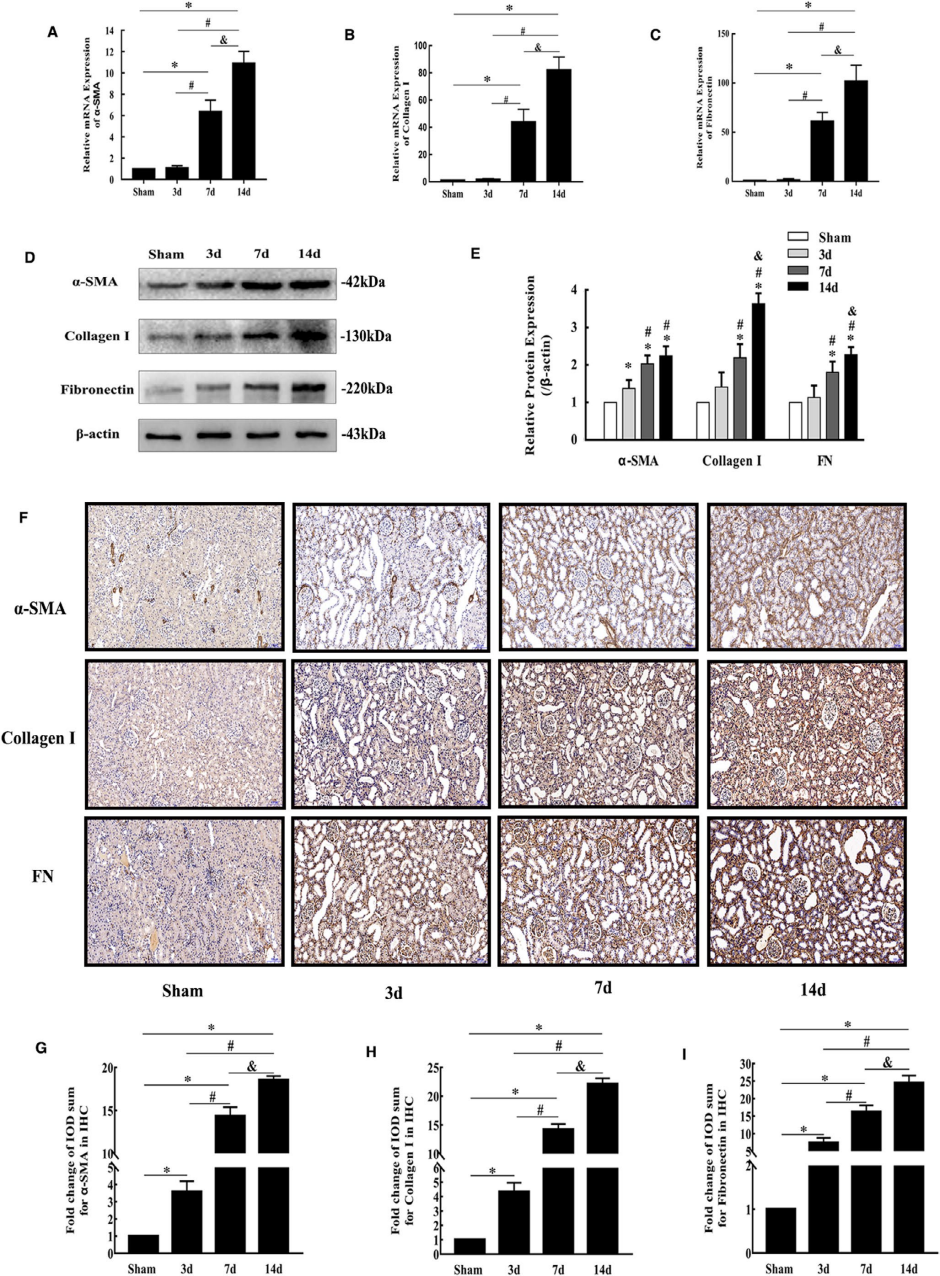
Progression of obstructive renal fibrosis in mice. (A‐C) Relative α‐SMA, collagen I and FN mRNA expression in the sham and UUO mouse groups determined by qRT‐PCR (mean ± SD, n = 3). (D‐E) Representative bands and fold changes in α‐SMA, collagen I and FN protein expression in the sham and UUO mouse groups determined by Western blotting (mean ± SD, n = 3). (F‐I) Representative IHC images and fold changes for α‐SMA, collagen I and FN in the different groups (100×); scale bar represents 100 μm (mean ± SD, n = 6). *P < .05, compared to the sham group. ^#^ *P* < .05, compared to the 3‐day UUO group. ^&^ *P* < .05, compared to the 7‐day UUO group. To simplify the figure, we only listed the data and representative pictures of sham‐operated mice killed on the 7th day after surgery to represent the sham group

According to the qRT‐PCR results, the mRNA expression levels of α‐SMA, collagen I and FN increased significantly in the 7‐day and 14‐day groups (Figure 3A‐C). Western blotting and immunohistochemical staining revealed that the three fibrosis indicators were up‐regulated in a time‐dependent manner, which further confirmed the development of fibrosis in obstructed kidneys in mice (Figure 3D‐I).

### 3.4 miR‐21‐5p promotes inflammation by activating the SPRY1/ERK/NF‐kB signalling pathway during obstructive renal fibrosis development

**FIGURE 4:**
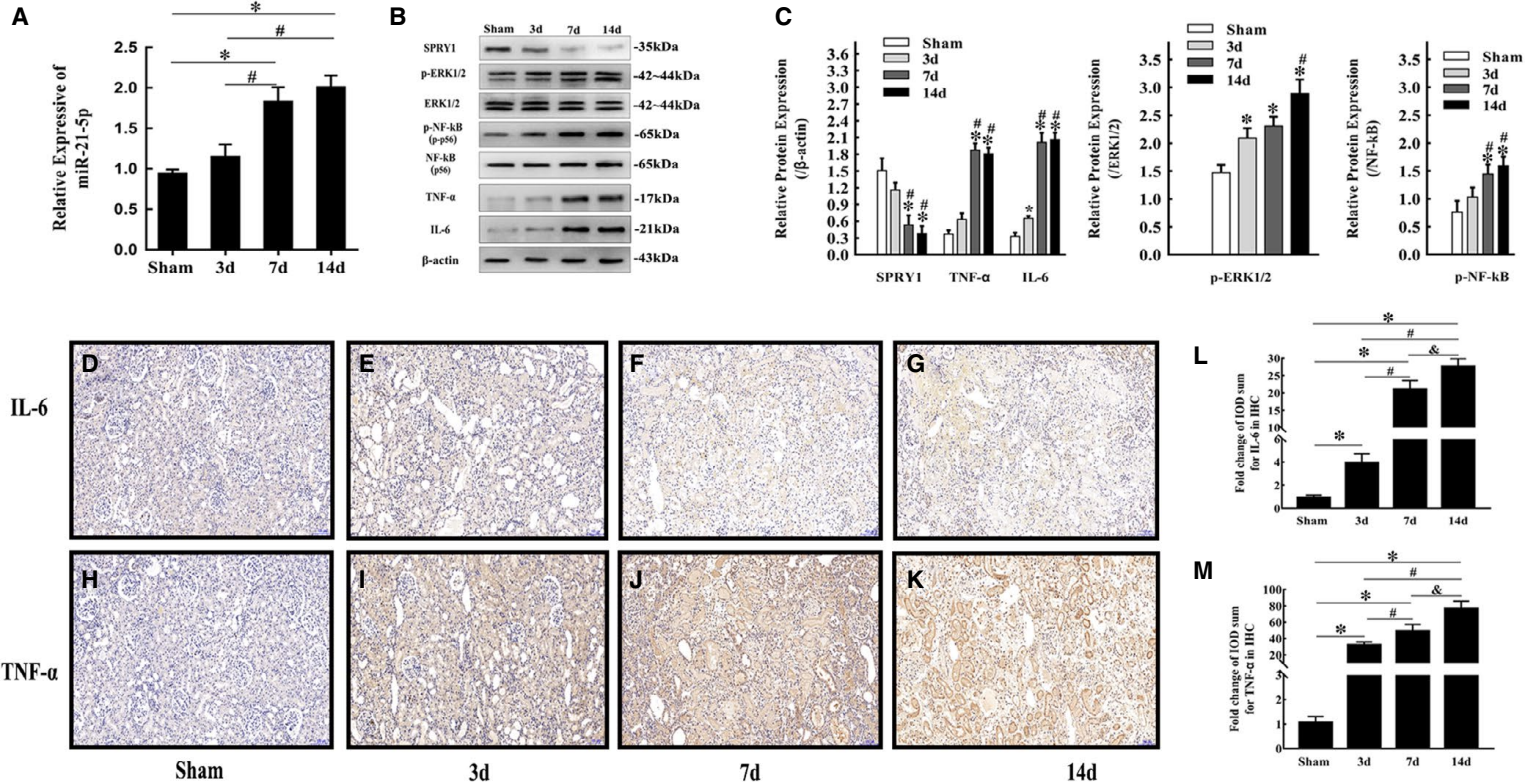
miR‐21‐5p up‐regulation in obstructed kidneys of mice activated the SPRY1/ERK/NF‐kB signalling pathway and inflammation. (A) miR‐21‐5p levels in obstructed kidneys of mice in different groups determined by qRT‐PCR (mean ± SD, n = 3). (B and C) Representative bands and fold changes in Spry1, p‐ERK1/2, ERK1/2, p‐NF‐κB, NF‐κB, IL‐6 and TNF‐α protein expression in obstructed kidneys of mice in different groups, determined by Western blotting (mean ± SD, n = 3). (D‐M) Representative IHC images and fold changes in IL‐6 and TNF‐α levels in different groups (100×); scale bar represents 100 μm (mean ± SD, n = 6).*P < .05, compared to the sham group. ^#^ *P* < .05, compared to the 3‐day UUO group. ^&^ *P* < .05, compared to the 7‐day UUO group. To simplify the figure, we only listed the data and representative pictures of sham‐operated mice killed on the 7th day after surgery to represent the sham group

**FIGURE 5:**
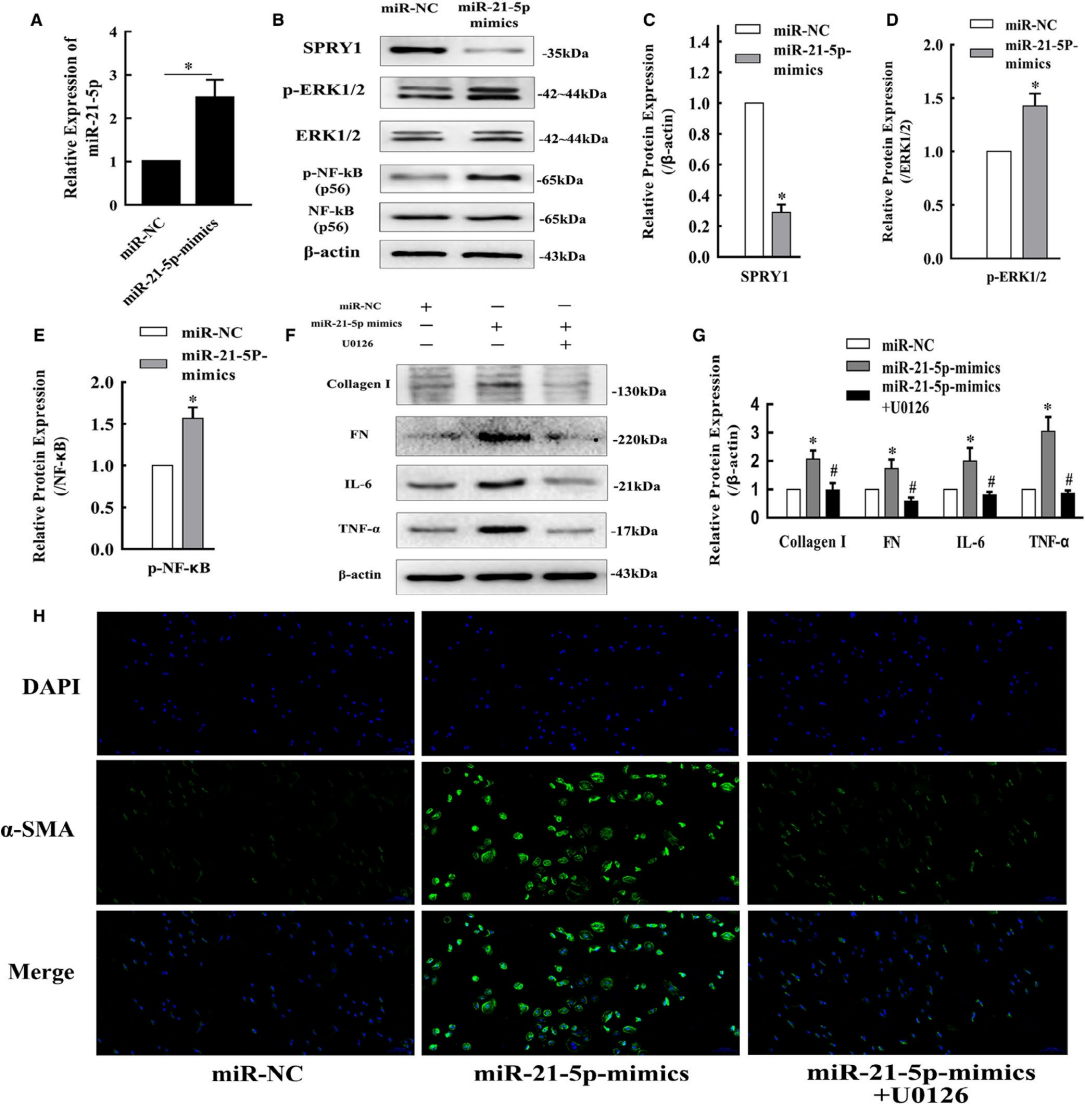
Enhanced miR‐21‐5p expression in HK‐2 cells promoted inflammation and fibrosis development via the SPRY1/ERK/NF‐kB signalling pathway. A, The fold change in miR‐21‐5p levels in HK‐2 cells transfected with miR‐21‐5p‐mimic determined by qRT‐PCR (mean ± SD, n = 3). (B‐E) Representative bands and fold changes in Spry1, p‐ERK1/2 and p‐NF‐κB protein expression in HK‐2 cells transfected with miR‐21‐5p‐mimic, determined by Western blotting (mean ± SD, n = 3). (F and G) Representative bands and fold changes in collagen I, FN, IL‐6 and TNF‐α protein expression in HK‐2 cells transfected with miR‐21‐5p‐mimic or treated with U0126, determined by Western blotting (mean ± SD, n = 3). H, Immunofluorescence staining of α‐SMA (100×) in HK‐2 cells showing that enhanced miR‐21‐5p expression increased α‐SMA expression, whereas treatment with U0126 reversed this effect; scale bar represents 100 μm. *P < .05, compared to miR‐NC. ^#^ *P* < .05, compared to miR‐21‐5p‐mimics

In our study, qRT‐PCR analysis showed that the miR‐21‐5p level increased significantly in a time‐dependent manner after ligation of the left ureter in mice (Figure 4A), which was accompanied by increased p‐ERK1/2, p‐NF‐κB, IL‐6 and TNF‐α protein levels and decreased SPRY1 protein levels (Figure 4B,C). We increased the miR‐21‐5p level in HK‐2 cells via transfection with a pGV514‐miR‐21‐5p‐mimic plasmid (Figure 5A). The increase in miR‐21‐5p promoted collagen I and FN synthesis and up‐regulated p‐ERK1/2, p‐NF‐κB, IL‐6 and TNF‐α protein expression in HK‐2 cells, while the expression of SPRY1 was inhibited (Figure 5B‐G). In contrast, treatment of HK‐2 cells with U0126, a specific ERK1/2 inhibitor, suppressed the collagen I, FN, IL‐6 and TNF‐α protein synthesis induced by transfection with miR‐21 mimic (Figure 5F,G). Moreover, immunofluorescence staining showed that α‐SMA expression was up‐regulated by miR‐21‐5p mimic, and this effect was reversed by U0126 (Figure 5H).

### 3.5 m^6^A methylation is significantly increased in obstructed kidney tissue, and the increased expression of METTL3, a methylation enzyme involved in m^6^A formation, plays a major catalytic role in obstructed kidneys

**FIGURE 6:**
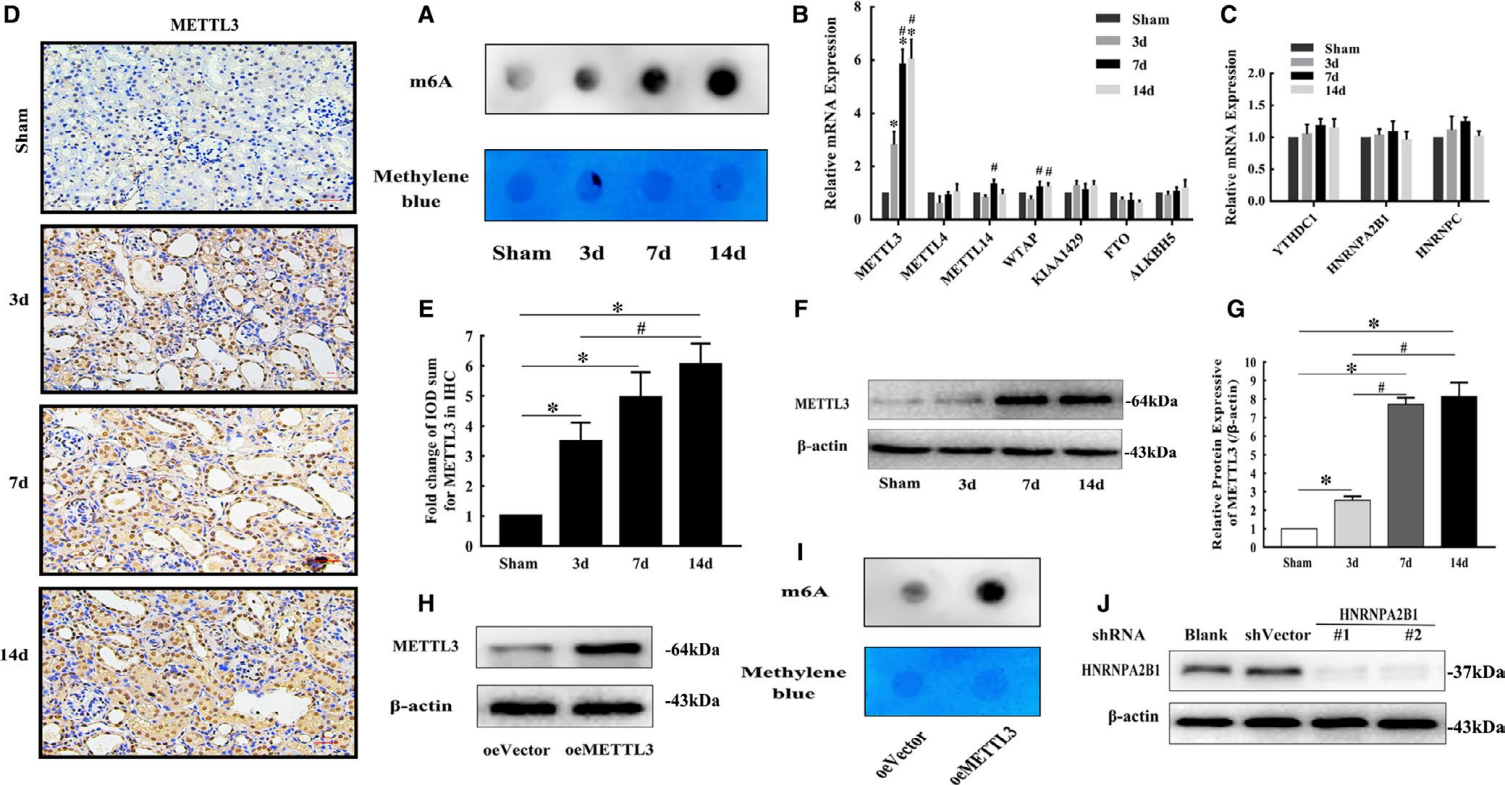
m^6^A methylation levels in RNA were increased in the UUO mouse groups, and METTL3 was the main functional methylation‐related enzyme. A, m^6^A dot blot assays of kidney tissues from mice in different groups. The methylation of RNA increased significantly in the UUO groups. Methylene blue stain was used as the loading control. B, mRNA levels of methylation enzymes involved in m^6^A formation in kidneys from mice in different groups (mean ± SD, n = 3). C, mRNA levels of nuclear reader proteins of m^6^A in kidneys from mice in different groups (mean ± SD, n = 3). (D and E) Representative IHC images and fold changes in METTL3 in kidneys from different groups (200×); scale bar represents 50 μm (mean ± SD, n = 6). (F and G) Representative bands and fold changes in METTL3 protein expression in kidneys from mice in different groups, determined by Western blotting (mean ± SD, n = 3). H, Representative bands of METTL3 protein expression in HK‐2 cells transfected with pGV657‐METTL3 plasmid, determined by Western blotting. I, m^6^A dot blot assays of HK‐2 cells. The methylation of RNA increased significantly after METTL3 overexpression. Methylene blue stain was used as the loading control. J, Representative bands of HNRNPA2B1 protein expression in HK‐2 cells transfected with PGV102‐shHNRNPA2B1#1 plasmid and PGV102‐shHNRNPA2B1#2 plasmid, demonstrated by Western blotting. *P < .05, compared to the sham mouse group or oeVector HK‐2 cell group. ^#^ *P* < .05, compared to the 3‐day UUO group. ^&^ *P* < .05, compared to the 7‐day UUO group. To simplify the figure, we only listed the data and representative pictures of sham‐operated mice killed on the 7th day after surgery to represent the sham group

The results of RNA m^6^A dot blot assays showed that m^6^A levels significantly increased after ligation of the left ureter for 3, 7 and 14 days (Figure 6A). The expression levels of key m^6^A methyltransferase and demethylase genes were detected via qRT‐PCR. The results showed that the METTL3 mRNA expression level was significantly increased in the UUO groups after ligation for 3, 7 and 14 days compared with the sham group, which was further confirmed by western blotting and IHC (Figure 6B,D‐G). The levels of METTL14, WTAP and other genes were also altered, but there was no significant difference compared with levels in the sham group. HK‐2 cells were transfected with the pGV657‐METTL3 plasmid to force METTL3 expression. The METTL3 protein level was increased after transfection compared to that in the control group (Figure 6H). As expected, overexpression of METTL3 also increased the global m^6^A modification level in HK‐2 cells (Figure 6I).

### 3.6 METTL3 may drive obstructive renal fibrosis development by promoting miR‐21‐5p maturation in obstructive renal fibrosis

**FIGURE 7:**
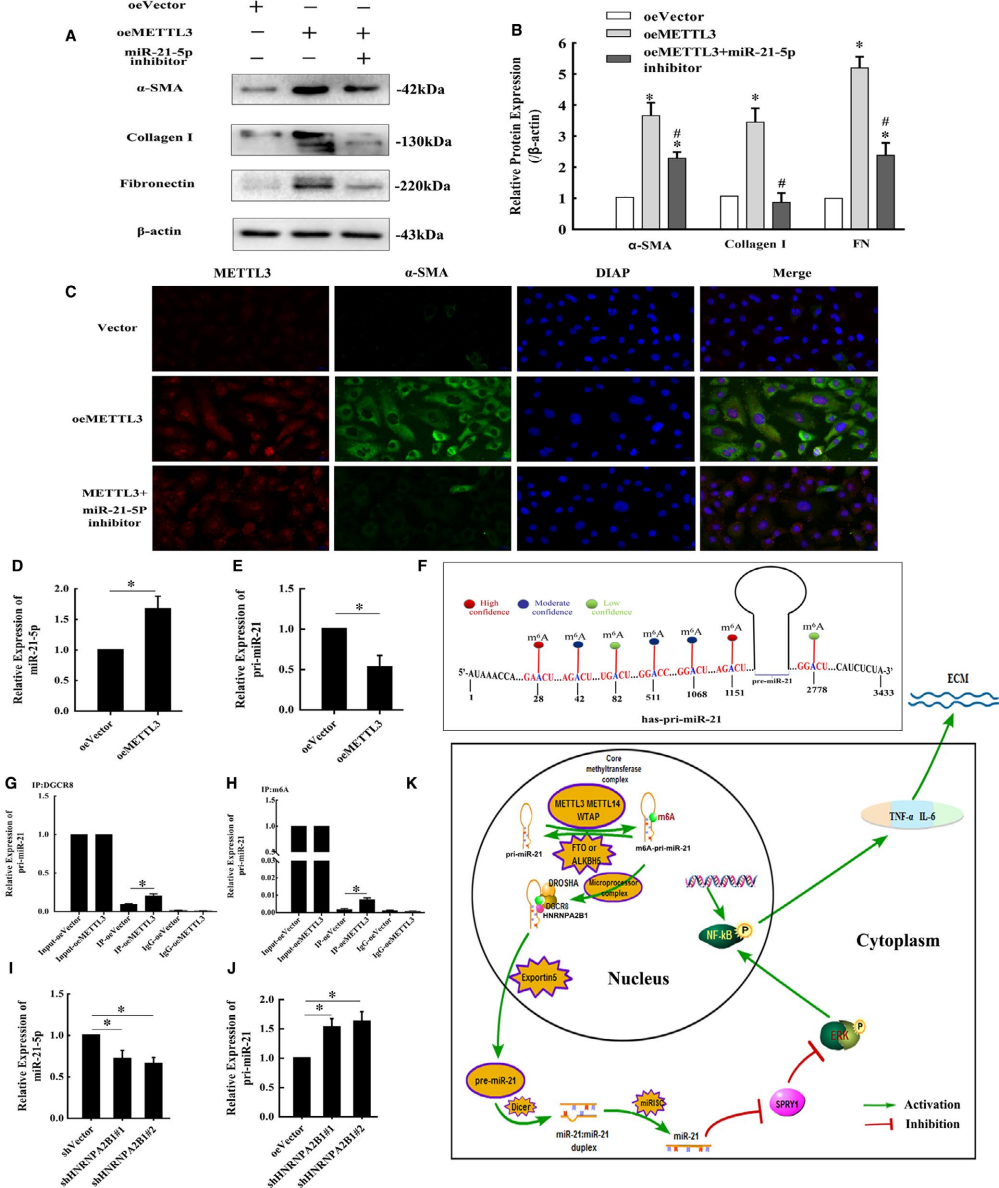
METTL3 overexpression in HK‐2 cells may drive fibrosis development by promoting miRNA‐21 maturation. (A and B) Representative bands and fold changes in α‐SMA, collagen I and FN protein expression in HK‐2 cells transfected with pGV657‐METTL3 plasmid or cotransfected with miR‐21‐5p inhibitor, demonstrated by Western blotting (mean ± SD, n = 3). C, Immunofluorescence staining of α‐SMA and METTL3 (400×) in HK‐2 cells showing that METTL3 enhancement increased α‐SMA expression, whereas cotransfection with the miR‐21‐5p inhibitor weakened this effect; scale bar represents 20 μm. (D and E) miR‐21‐5p and pri‐miR‐21 levels in HK‐2 cells transfected with pGV657‐METTL3 plasmid determined by qRT‐PCR (mean ± SD, n = 3). F, Seven possible m^6^A modification sites exist in hsa‐pri‐miR‐21, including 2 high confidence positions based on prediction with SRAMP. G, The levels of pri‐miRNA‐21‐5p binding to DGCR8 in HK‐2 cells with METTL3 up‐regulation determined by qRT‐PCR. H, Immunoprecipitation of m^6^A‐modified RNA in HK‐2 cells with METTL3 up‐regulation, followed by qRT‐PCR to assess the pri‐miR‐21 m^6^A modification level. (I and J) miR‐21‐5p and pri‐miR‐21 levels in HK‐2 cells transfected with PGV102‐shHNRNPA2B1#1 plasmid and PGV102‐shHNRNPA2B1#2 plasmid measured via qRT‐PCR (mean ± SD, n = 3). K, Mode pattern of the METTL3‐m^6^A‐miR‐21‐5p‐SPRY1/ERK/NF‐kB regulatory network in obstructive renal fibrosis *P < .05, compared to the oeVector group. ^#^ *P* < .05, compared to the oeMETTL3 group

In the in vivo study, METTL3 was significantly up‐regulated in the UUO groups, accompanied by a time‐dependent increase in mature miR‐21‐5p (Figure 4A). Overexpression of METTL3 in HK‐2 cells significantly promoted the expression of fibrosis indicators, including α‐SMA, collagen I and FN, according to the Western blotting and immunofluorescence staining results, while the pro‐fibrotic effect was obviously weakened by cotransfection with the miR‐21‐5p inhibitor (Figure 7A,B,C). Moreover, qRT‐PCR analysis revealed that mature miR‐21‐5p was increased and unprocessed pri‐miR‐21 was decreased in METTL3‐overexpressing HK‐2 cells (Figure 7D,E). The sequence of hsa‐pri‐miR‐21 was downloaded from Ensembl database. Then, the m^6^A modification sites were predicted via SRAMP (http://www.cuilab.cn/sramp/), which is a sequence‐based m^6^A modification site predictor. We found 7 positions in hsa‐pri‐miR‐21 that may be m^6^A modification sites, and the prediction score for 2 of the positions was distributed in the high confidence range (Figure 7F). The results of a RIP assay with anti‐DGCR8 antibody revealed that the level of pri‐miR‐21 binding to DGCR8 was significantly increased in the HK‐2 cells with up‐regulated METTL3 (Figure 7G). Furthermore, as expected, a RIP assay performed with m^6^A antibody revealed that the level of m^6^A‐modified pri‐miR‐21 was elevated in the HK‐2 cells with METTL3 up‐regulation (Figure 7H).

### 3.7 HNRNPA2B1 may act as a reader protein of METTL3‐mediated m^6^A in pri‐miR‐21 and promote maturation of miR‐21‐5p

The recognition of m^6^A in pri‐miRNAs by reader proteins occurs in the nucleus; thus, the possible reader proteins of m^6^A in pri‐miR‐21, including YTHDC1, HNRNPA2B1 and HNRNPC, were evaluated via qRT‐PCR, but the results did not reveal any obvious changes (Figure 6C). Then, HK‐2 cells were transfected with the PGV102‐shHNRNPA2B1#1 plasmid and PGV102‐shHNRNPA2B1#2 plasmid to inhibit HNRNPA2B1 expression. The protein levels of HNRNPA2B1 were decreased after transfection (Figure 6J). Then, qRT‐PCR analysis revealed that mature miR‐21‐5p was decreased and pri‐miR‐21 was accumulated in HNRNPA2B1‐knockdown HK‐2 cells (Figure 7I,J).

Taken together, our results showed that up‐regulation of METTL3 in the obstructed kidneys of mice can increase m^6^A modification of pri‐miR‐21, promote maturation of miRNA‐21‐5p and then activate the downstream SPRY1/ERK/NF‐κB signalling pathway to drive inflammation and obstructive renal fibrosis development. Moreover, HNRNPA2B1 may take part in recognition of METTL3‐mediated m^6^A in pri‐miR‐21 and promote maturation of miR‐21‐5p (Figure 7K).

## 4 DISCUSSION

Fibrosis is a pathological characteristic of the later stage of hydronephrosis. ECM deposition damages the normal structure of renal tubules and glomeruli and inhibits renal function. To date, it is difficult to reverse renal fibrosis using existing treatments. Deeper exploration of the mechanism of fibrosis progression in obstructed kidneys is essential to find a breakthrough treatment. In our study, we established a UUO model in mice to simulate the pathological process of obstructive renal fibrosis and investigated the molecular mechanism through in vitro experiments using HK‐2 cells. We revealed that m^6^A‐dependent pri‐miR‐21 processing promoted miR‐21‐5p expression and impacted obstructive renal fibrosis by activating the SPRY1/ERK/NF‐κB pathway. To the best of our knowledge, this is the first time that the METTL3‐m^6^A‐miR‐21‐5p‐SPRY1/ERK/NF‐kB axis has been demonstrated to play a role in obstructive renal fibrosis.

It has been well documented that miR‐21 is involved in fibrosis of the kidneys and other organs, including the heart,^21^ liver^22^ and lungs.^23^ According to previously published studies,^24^, ^25^ the miR‐21 level is increased in renal fibrosis tissue and serves as an important biomarker of fibrosis in plasma^26^ and urinary tissue.^27^ Ample evidence supports miR‐21 as a promoting factor during renal fibrosis, and targeting miR‐21 likely has a therapeutic effect and attenuates renal fibrosis development.^28^, ^29^ In our study, after ligation of the left ureter for 7 and 14 days, the morphology of obstructed kidneys changed dramatically. The SCr and BUN levels showed an upward trend within the normal range, which was related to the extent of impairment of the left kidney and was consistent with the results of a previous study.^30^ The expression of fibrosis indicators, including α‐SMA, collagen I and FN, was significantly elevated and the miR‐21‐5p level was significantly increased in the 7‐day and 14‐day groups, which was consistent with previously reported studies. miR‐21 exerts pro‐fibrotic effects via complex downstream signalling pathways.^5^ The mitogen‐activated protein kinase (MAPK) signalling pathway is well reported and is strongly associated with fibrosis progression.

ERK is a member of the MAPK family and has important roles in many signalling cascades. The ERK signalling pathway participates in the fibrotic process in many organs, including the artery,^31^ ligamentum flavum,^32^ liver,^33^ lungs^34^ and kidneys.^35^ Phosphorylated ERK1/2 (p‐ERK1/2) can activate NF‐κB, and phosphorylated NF‐κB (p‐NF‐κB) translocates into the nucleus to bind to specific DNA fragments and regulate inflammation‐related gene and protein expression, which is crucial for initiation and progression of inflammation in many diseases, including renal fibrosis.^36^

Recently, Ning et al^7^ and Sun et al^8^ reported that miR‐21 mediates angiotensin II–induced liver and pulmonary fibrosis via the ERK/NF‐κB pathway by directly targeting SPRY1. However, thus far, the role of the interaction between miR‐21 and the ERK/NF‐κB pathway in obstructive renal fibrosis has not been reported. In the UUO groups in our study, p‐ERK1/2 and p‐NF‐κB protein expression was up‐regulated, while the expression of SPRY1 was inhibited. Infiltration of inflammatory cells was observed, and inflammation foci were formed in the 7‐day and 14‐day groups. The TNF‐α and IL‐6 protein levels were significantly increased. Masson's trichrome staining showed higher collagen deposition in areas of inflammation foci. Therefore, we propose that the pro‐fibrotic effect of miR‐21‐5p in obstructive nephropathy may be associated with activation of ERK/NF‐κB signalling and inflammation. Overexpression of miR‐21‐5p induced by transfection with miR‐21‐5p mimic in HK‐2 cells significantly increased the p‐ERK, p‐NF‐κB, collagen I, FN, IL‐6 and TNF‐α protein levels and decreased the SPRY1 protein levels. Moreover, the up‐regulation of collagen I, FN, IL‐6 and TNF‐α expression induced by transfection with miR‐21‐5p mimic was reversed by U0126, which is a specific ERK1/2 inhibitor. Thus, our data indicate that the increased miR‐21‐5p level promoted inflammation in obstructive nephropathy by activating the SPRY1/ERK/NF‐kB signalling pathway and contributed to obstructive renal fibrosis development.

Accumulating evidence has demonstrated that m^6^A methylation is essential for regulating microRNA metabolism during many biological processes.^18^, ^37^, ^38^, ^39^, ^40^, ^41^ Zhang et al^37^ reported that m^6^A methylation up‐regulated by cigarette smoke condensate resulted in excessive miR‐25‐3p maturation and promoted pancreatic cancer progression. Han et al^18^ demonstrated that a reduction in m^6^A methylation led to down‐regulation of miRNA‐126 and promoted fibrosis in the lung after CB exposure. In the present study, we found that m^6^A levels significantly increased in the UUO groups, accompanied by a time‐dependent increase in mature miR‐21‐5p. Thus, we speculate that the enhanced miR‐21‐5p expression in UUO mouse models may be caused by altered m^6^A methylation.

Research on the role of m^6^A methylation in obstructive renal fibrosis development is very limited. In March of 2020, Liu et al^42^ reported that the m^6^A level increased in HK2 cells treated with TGF‐β1 and that METTL3, METTL14 and WTAP were up‐regulated, suggesting that m^6^A methylation occurs in renal fibrogenesis. In July of 2020, Li X et al^30^ revealed that total m^6^A levels in the kidney were time‐dependently decreased within 1 week after UUO establishment in mice but slightly rebounded at day 14. Thus, the authors concluded that m^6^A has functional importance in renal interstitial fibrosis during obstructive nephropathy and might be a promising therapeutic target. In November of 2020, Ning et al^43^ reported that the total m^6^A level increased significantly at day 7 after left ureter ligation in mice, and the authors believed that genistein ameliorates renal fibrosis by reducing RNA m^6^A levels in UUO model mice. The total levels of m^6^A in UUO‐induced renal fibrosis have shown the opposite trend in different studies, and the dynamic and reversible m^6^A methylation process might explain this to some extent. A similar conflict in reported results also occurs in the expression of methylation‐related enzymes.

A previous study proposed that METTL3 is the catalytic subunit of the core methyltransferase complex, METTL14 provides structural support for METTL3, and WTAP stabilizes the core complex.^44^ However, other studies have reached different conclusions. The results of Ma et al^40^ suggested that METTL14 is responsible for aberrant m^6^A methylation in hepatocellular carcinoma. Zhuang et al^45^ reported that low expression of obesity‐associated protein (FTO) in human clear cell renal cell carcinoma causes up‐regulation of m^6^A and is correlated with increased tumour severity and poor patient survival. Accordingly, altered m^6^A methylation may be caused by different methylation‐related enzymes in different biological processes.

In our in vivo study, among the tested methylation‐related enzymes, METTL3 was the most highly up‐regulated in the UUO mouse groups. In the in vitro study, overexpression of METTL3 in HK‐2 cells resulted in up‐regulation of m^6^A methylation. Thus, among the methylation‐related enzymes involved in m^6^A formation, METTL3 might play the main functional role in obstructed kidneys in mice. Moreover, enhanced METTL3 in HK‐2 cells increased the miR‐21‐5p expression level and the protein expression of fibrosis indicators, including α‐SMA, collagen I and FN, but the expression of pri‐miR‐21 was decreased. Moreover, the pro‐fibrotic effect of METTL3 overexpression in HK‐2 cells was alleviated by cotransfection with a miR‐21‐5p inhibitor. Therefore, METTL3 may drive obstructive renal fibrosis development by promoting miR‐21‐5p maturation.

RIP assays revealed that the contents of m^6^A‐modified pri‐miR‐21 and the levels of pri‐miR‐21 binding with DGCR8 were significantly increased in HK‐2 cells with METTL3 up‐regulation. A previous study^46^ reported the sequence of the full‐length, ∼3433‐nt hsa‐pri‐miR‐21 RNA, and 7 possible m^6^A modification sites exist in hsa‐pri‐miR‐21, including 2 high confidence positions based on prediction with SRAMP. In addition, according to an important study^17^ published in CELL in 2015, HNRNPA2B1 is a reader protein of m^6^A modification sites in pri‐miRNAs and has similar effects on alternative splicing as METTL3 modulation. In the study, HNRNPA2B1 depletion in MDA‐MB‐231 and HEK293 cells caused a reduction in the expression level of mature miRNAs and resulted in accumulation of specific pri‐miRNAs in the nucleus. In the present study, we knocked out HNRNPA2B1 in HK‐2 cells and found similar results: The expression level of miR‐21‐5p decreased, and pri‐miR‐21 accumulated in the cells. Therefore, HNRNPA2B1 might be a reader protein of m^6^A in pri‐miR‐21 and recruit DGCR8 for processing. These results confirm that METTL3 mediates m^6^A up‐regulation in UUO mice and indeed enhances miR‐21‐5p maturation by m6A‐dependent pri‐miR‐21 processing, thus driving obstructive renal fibrosis development.

## 5 CONCLUSION

In summary, our research revealed a significant increase in m^6^A modification levels and miR‐21‐5p expression in obstructed kidneys of mice. Among the enzymes that regulate m^6^A modification, METTL3 might play the main functional role. Furthermore, METTL3 catalysis of m^6^A modification may drive obstructive renal fibrosis development by promoting miR‐21‐5p maturation in mice. We also found that miR‐21‐5p promoted inflammation in obstructed kidneys by activating the SPRY1/ERK/NF‐kB signalling pathway and ultimately contributed to obstructive renal fibrosis progression. Our results might help deepen understanding of the role of m^6^A modification and miR‐21‐5p in obstructive renal fibrosis. The identified METTL3‐m^6^A‐miR‐21‐5p‐SPRY1/ERK/NF‐kB axis provides new insight into the pathogenesis of obstructive renal fibrosis. In our ongoing follow‐up studies, we intend to continue this research and further explore the therapeutic role of this axis in obstructive renal fibrosis.

## CONFLICT OF INTEREST

The authors confirm that there are no conflicts of interest.

## AUTHOR CONTRIBUTIONS

**Erpeng Liu:** Conceptualization (lead); Data curation (lead); Formal analysis (lead); Methodology (lead); Project administration (lead); Software (lead); Visualization (lead); Writing‐original draft (lead). **Lei** **Lv:** Data curation (supporting); Formal analysis (supporting); Visualization (supporting). **Yonghao Zhan:** Conceptualization (supporting); Data curation (supporting). **Yuan Ma:** Conceptualization (supporting); Writing‐original draft (supporting). **Jinjin Feng:** Conceptualization (supporting); Writing‐review & editing (supporting). **Yulin He:** Conceptualization (supporting); Visualization (supporting). **Yibo Wen:** Data curation (supporting); Formal analysis (supporting). **Yanping Zhang:** Data curation (supporting); Formal analysis (supporting); Software (supporting). **Qingsong Pu:** Data curation (supporting); Formal analysis (supporting); Visualization (supporting). **Fengping Ji:** Data curation (supporting); Formal analysis (supporting); Visualization (supporting). **Xinghuan Yang:** Data curation (supporting); Formal analysis (supporting); Visualization (supporting). **Jian** **Guo Wen:** Conceptualization (lead); Data curation (lead); Formal analysis (lead); Funding acquisition (lead); Investigation (supporting); Methodology (supporting); Project administration (lead); Resources (lead); Software (supporting); Supervision (lead); Validation (supporting); Visualization (supporting); Writing‐original draft (lead); Writing‐review & editing (lead).

## Supporting information

Supplementary Material

## Data Availability

The data that support the findings of this study are available from the corresponding author upon reasonable request.
