# Supplementary material for: METTL3/N6‐methyladenosine/ miR‐21‐5p promotes obstructive renal fibrosis by regulating inflammation through SPRY1/ERK/NF‐κB pathway activation
Source: J Cell Mol Med. 2021 Jun 24;25(16):7660–74. doi: 10.1111/jcmm.16603 (PMC8358893; doi:10.1111/jcmm.16603)
Supplement: Supplementary file 1 — Supplementary Material [file JCMM-25-7660-s001.docx]

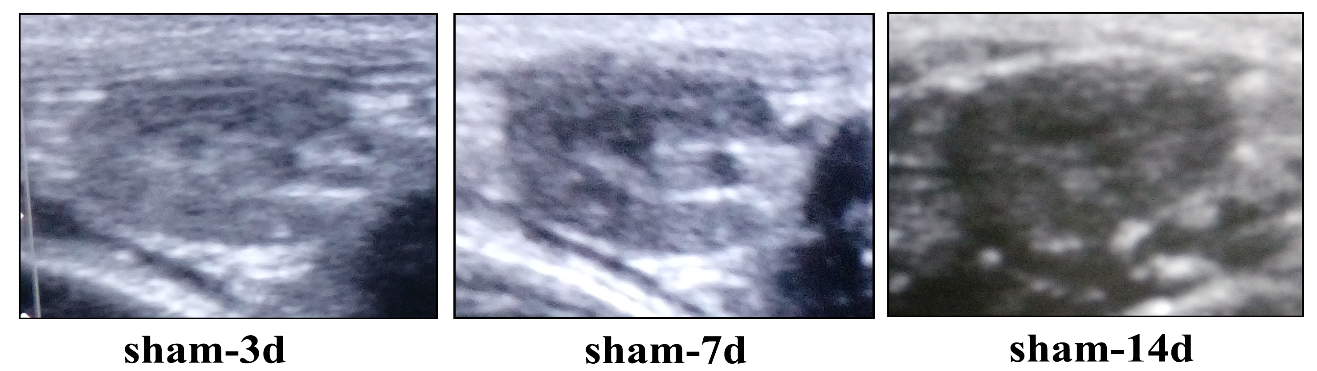


**Supplemental figure.1**

Ultrasonography revealed that the left kidneys of sham-operated mice had no hydronephrosis at three time points for sacrifice.


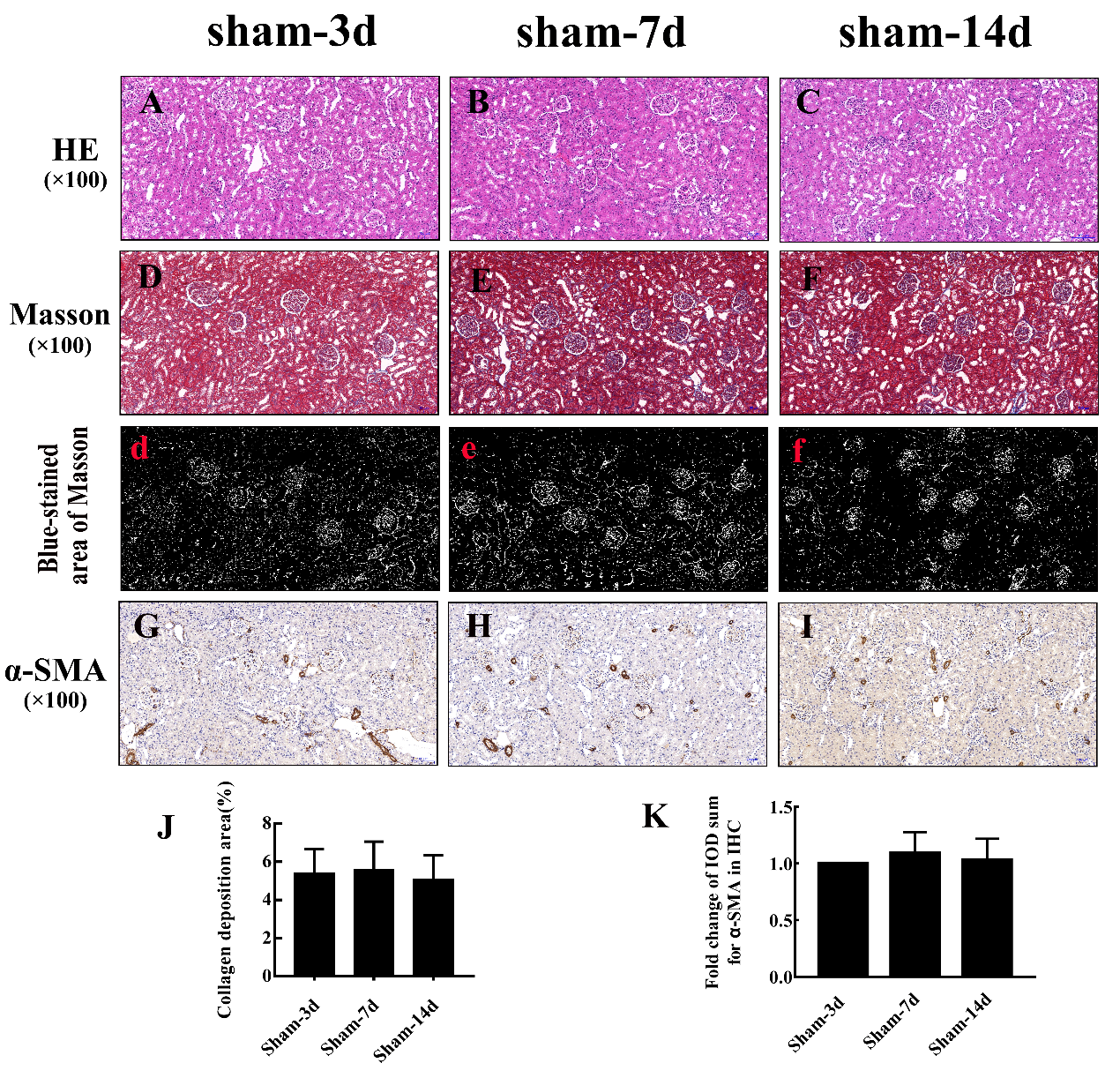


**Supplemental figure.2**

The results of HE staining, Masson’s trichrome staining and IHC of α-SMA in Sham group at three execution time points have no significant difference.

(A-C) Representative histopathology images of sham operated kidneys in mice by HE staining (100×). Scale bars represent 100 μm. (D-F) Representative histopathology images of sham operated kidneys in mice by Masson’s trichrome staining (100×). Scale bar represents 100 μm. (d-f) Blue-stained areas of Masson’s trichrome staining, which were transformed from image D, E and F by Image-Pro Plus 6.0 software. The white area refers to collagen deposition area. (G-I) Representative IHC images and α-SMA in different groups (100×); scale bar represents 100 μm (mean ± SD, n=6). (J) The statistical analysis of the degrees of collagen deposition in sham group at three execution time points has no significant difference (mean ± SD, n=6). (K) Fold change of IOD sum for IHC of α-SMA in sham group at three execution time points has no significant difference (mean ± SD, n=6)

**The sequence of the full-length, ∼3433-nt has-pri-miR-21 RNA from Ensembl database.**

>17 dna:chromosome chromosome:GRCh38:17:59838822:59842255:1

AUAAACCAAGGCUCUUACCAUAGCUGAACUUUAAAACUUAGACUGUCUUUUCUGUAAACGAUUCUGAGGCAAAGGGAAAUGACUAGAAGAGGAUGAGUAAACAAUAACCUGAAAUGGGAAACUCGAGGGAAGCACAGGUUUUUUUUGUUUUGUUUUGUUUGGUUCGUUUUUUGUUCUUUGGGGUUUUUUUGAGACAGAAUUUCGCUCUCGUUGCCCAAGUUGGAGUGCAAUGGCGCGAUCUUGGCUCACUGCAACCUCCGCCUCCCGGGUUCAAGCGAUUCUCCUGCCUCAGCCUCCCAAGUAGCUGUGAUUCCAGGCACGUGCCACCACACCAGCUAAUUUUUUGUAUUUUAAUAGAAACAGGGUUUCACCGUGUUAGCCAGGCUGGUCUCAAACUGACCUCAGAUGAUCCGCCCGCCUUGGCCUCCCAAAGUGCUGGGAUUACAGAUGUGAGCCACCGCGCCCGGCCAGAGCACUGUUUUUUUUAAUGGCCUUGCACUCUUCUUAUGGACCUUUGCUGCCCUCAGUUGACCAAACAUGACAUCAGAAACAGAUACAUUUGUGUGUUUUAAAAACAGCUCCUAAUACUGGAACAAAAAUAUUUAACUGUCUUGACAAUACUCAUGAGUAUCUGCAUGGCGACUUCAGAGUUGAGUUUAAUCAAAGAGUUUAUUCUUAGGUCCUAGUAGAAGAGCUAACCUCACACUCAUCCCAUUCUAAACUAUGUGAUUCAACACUGAUUUUACAUCCAACAAAGUGAAAUCUUGAUAGUUGGGUGUAAAAAGGAGAGUAAUGGAGAUUUCAGAGUAGUUGGGGUUGCUUACUUUUCAUUUUUAAUUCUUUAGGUUUUGUAAGUUACACACUUCAAGCAUUAUAGAUGAUCCUCUUUUUACUACUGAACUAAUGAAGCCUUUUUCAUUGCAUUGUUCUGCAUUUAUUUCUACAGGGAGAAAACUGGUUGUCCUGGAUGUUUGAAAAGUUGGUCGUUGUCAUGGUGUGUUACUUCAUCCUAUCUAUCAUUAACUCCAUGGCACAAAGUUAUGCCAAACGAAUCCAGCAGCGGUUGAACUCAGAGGAGAAAACUAAAUAAGUAGAGAAAGUUUUAAACUGCAGAAAUUGGAGUGGAUGGGUUCUGCCUUAAAUUGGGAGGACUCCAAGCCGGGAAGGAAAAUUCCCUUUUCCAACCUGUAUCAAUUUUUACAACUUUUUUCCUGAAAGCAGUUUAGUCCAUACUUUGCACUGACAUACUUUUUCCUUCUGUGCUAAGGUAAGGUAUCCACCCUCGAUGCAAUCCACCUUGUGUUUUCUUAGGGUGGAAUGUGAUGUUCAGCAGCAAACUUGCAACAGACUGGCCUUCUGUUUGUUACUUUCAAAAGGCCCACAUGAUACAAUUAGAGAAUUCCCACCGCACAAAAAAAGUUCCUAAGUAUGUUAAAUAUGUCAAGCUUUUUAGGCUUGUCACAAAUGAUUGCUUUGUUUUCCUAAGUCAUCAAAAUGUAUAUAAAUUAUCUAGAUUGGAUAACAGUCUUGCAUGUUUAUCAUGUUACAAUUUAAUAUUCCAUCCUGCCCAACCCUUCCUCUCCCAUCCUCAAAAAAGGGCCAUUUUAUGAUGCAUUGCACACCCUCUGGGGAAAUUGAUCUUUAAAUUUUGAGACAGUAUAAGGAAAAUCUGGUUGGUGUCUUACAAGUGAGCUGACACCAUUUUUUAUUCUGUGUAUUUAGAAUGAAGUCUUGAAAAAAACUUUAUAAAGACAUCUUUAAUCAUUCCAAAAUUGUGUCCGUUUUCUUGAGCGUUUUGAUUUUUUACUUUUAGCUUAUACCAGCUGAAUGGCAGCCUUGCCUAAUCCACCUACAACAAGAAUUUCUUAAGCUUUCUUUUAUUUGCAUGAGAGAGCCACUACCAAGGCAUGUUUUGUUAUGCUGAAACUGGGCUGCUGCAUACUGCUAAAUGGCACCUCUGGGAUUGGCCUACCUGGGGAUUUCUUGGUUUGUGAAAACAGGAGAGGAGAAAUAUCUCAUACAAGUGAAAGGAUACUGGAGAGAGAAAUUACCCAUUUCUAAAAAAAAACCACACUCUGUCGUAUCUGUGUUAAUGUUUUCUAGCAUGUACUCUGGUUUCAACAGACACAAAUUUAUAUGUUAACCCAGUUUUCUUGCCGUUCUGUAAGUGUUUUAUUCUUAGUGUGAUUUUUUUCCAUUGGGAUGUUUUUGAUUGAACUUGUUCAUUUUGUUUUGCUUGGGAGGAAAAUAAACAAUUUUACUUUUUUCCUUUAGGAGCAUUAUGAGCAUUAUGUCAGAAUAGAAUAGAAUUGGGGUUCGAUCUUAACAGGCCAGAAAUGCCUGGGUUUUUUUGGUUUGUUUUUGUUUUUGUUUUUUUAUCAAAUCCUGCCUGACUGUCUGCUUGUUUUGCCUACCAUCGUGACAUCUCCAUGGCUGUACCACCUUGUCGGGUAGCUUAUCAGACUGAUGUUGACUGUUGAAUCUCAUGGCAACACCAGUCGAUGGGCUGUCUGACAUUUUGGUAUCUUUCAUCUGACCAUCCAUAUCCAAUGUUCUCAUUUAAACAUUACCCAGCAUCAUUGUUUAUAAUCAGAAACUCUGGUCCUUCUGUCUGGUGGCACUUAGAGUCUUUUGUGCCAUAAUGCAGCAGUAUGGAGGGAGGAUUUUAUGGAGAAAUGGGGAUAGUCUUCAUGACCACAAAUAAAUAAAGGAAAACUAAGCUGCAUUGUGGGUUUUGAAAAGGUUAUUAUACUUCUUAACAAUUCUUUUUUUCAGGGACUUUUCUAGCUGUAUGACUGUUACUUGACCUUCUUUGAAAAGCAUUCCCAAAAUGCUCUAUUUUAGAUAGAUUAACAUUAACCAACAUAAUUUUUUUUAGAUCGAGUCAGCAUAAAUUUCUAAGUCAGCCUCUAGUCGUGGUUCAUCUCUUUCACCUGCAUUUUAUUUGGUGUUUGUCUGAAGAAAGGAAAGAGGAAAGCAAAUACGAAUUGUACUAUUUGUACCAAAUCUUUGGGAUUCAUUGGCAAAUAAUUUCAGUGUGGUGUAUUAUUAAAUAGAAAAAAAAAAUUUUGUUUCCUAGGUUGAAGGUCUAAUUGAUACGUUUGACUUAUGAUGACCAUUUAUGCACUUUCAAAUGAAUUUGCUUUCAAAAUAAAUGAAGAGCAGCUGUCCUUCUUUCCUCUUUUAAGUGUUCAGCUGUGGCAUGCUCAGAGGUUCCUGCUGGAUUCCAGCUGGAGCGGUGUGAUACCCUUCUUUUUCAGCUGUUCGUGCCUUCCUUUCUUGUAUCCACCAAAGUGGAGACAAAUACAUGAUCUCAAAGAUACACAGUACCUACUUAAUUCCAGCUGAUGGGAGACCAAAGAAUUUGCAAGUGGAUGGUUUGGUAUCACUGUAAAUAAAAAGAGGGCCUGGGAAUUCUUGCGAUUCCAUCUCUA
